# Osteoradionecrosis of the jaws triggered by dental implants placement: A case report

**DOI:** 10.4317/jced.55326

**Published:** 2019-01-01

**Authors:** Jorge Toledano-Serrabona, Gabriela Párraga-Manzol, Mª Ángeles Sánchez-Garcés, Cosme Gay-Escoda

**Affiliations:** 1 DDS. Dental Degree. Faculty of Medicine and Health Sciences, University of Barcelona, Barcelona (Spain); 2 DDS, MS. Master’s Degree Program in Oral Surgery and Implantology, Faculty of Medicine and Health Sciences, University of Barcelona (Spain); 3 MD, DDS, MS, PhD, EBOS. Aggregate Professor of Oral Surgery. Master’s Degree Program in Oral Surgery and Implantology, Faculty of Medicine and Health Sciences, University of Barcelona. Researcher of the IDIBELL institute, Barcelona (Spain); 4 MD, DDS, MS, PhD, EBOS, OMFS. Chairman and Professor of Oral and Maxillofacial Surgery, Faculty of Medicine and Health Sciences, University of Barcelona. Director of the Master’s Degree Program in Oral Surgery and Implantology (EFHRE International University/FUCSO). Coordinator/Researcher of the IDIBELL Institute. Head of the Oral Surgery, Implantology and Maxillofacial Surgery Department of the Teknon Medical Center, Barcelona (Spain)

## Abstract

**Background:**

The decision-making process about how to rehabilitate edentulous osseous defects in patients with head and neck cancer history can be complex. Even though, endosseous dental implants could be considered to be the first choice for treating these patients, it is highly important to be aware of the complications that might occur. The aim of this report was to describe the clinical features of mandibular fracture after dental implants placement on a cancer irradiated patient and update the available information about this event.

**Case report:**

The case describes a 70-year-old man, with medical background of radiotherapy in jaw bones to treat a carcinoma in the floor of the mouth and later on in the soft palate and cheek. One week after dental implant surgery, the patient presented a mandibular osteoradionecrosis that healed in 8 months. A fracture on the right side of the body mandible was diagnosed one year after implant placement. Although several options were suggested in order to repair the fracture, the patient did not accept any further treatment despite the callus formation not being radiographically evident. The implant-supported prosthesis is functionally useful for more than 8 years of follow-up without significant problems.

**Conclusions:**

The implant treatment and management of oncologic irradiated patients require special considerations due to the risk of osteoradionecrosis and its possible complications, such as pathologic fracture. It is necessary to provide full information to the patient about risk factors and complications.

** Key words:**Dental implants, mandibular fracture, osteoradionecrosis jaw, complications, fracture, cancer.

## Introduction

Mandibular defects caused by ablative tumor resection, bone atrophy or trauma may result in aesthetic deficits and functional impairments on the orofacial system. Oral rehabilitation with dental implants could improve the quality of life of these patients as it has been reported previously (1).

The most frequent biological complication with dental implants is the failure to achieve osteointegration (2). There are less common complications, such as the mandibular fracture or the osteoradionecrosis (ORN) specially in the irradiated jaws (3).

Patients having oncologic treatment are a challenging population with riskier than healthy subjects, especially if the cancer treatment plan also includes the irradiation of the jaw bones (4). The indication of implant-supported rehabilitations in irradiated patients have been evaluated in several studies and it could be concluded that dental implants are a valuable tool after oncologic treatment of the head and neck region, despite there being some risk factors such as age, gender, total radiation dose, period between the end of radiotherapy and implant surgery, and the type of radiation therapy that have to be considered in order to avoid complications (4–6).

The aim of this report was to describe the clinical features of mandibular fracture after dental implants placement on an irradiated patient performed according to “CAse REport” (CARE) guidelines (7) and update the information available about this type of event.

## Case Report

In July 2008, a 65-year-old caucasian man completely edentulous came to the Oral Surgery and Implantology Department of the University of Barcelona (Barcelona, Spain) to evaluate his possibilities of oral rehabilitation.

Patient pathological background included: smoke habit of 60 cigarettes per day for 30 years until 1999 and alcohol consumption of 150 gr/day (cessation in 1999), Oral Squamous Cell Carcinoma (OSCC) in the right side of the floor of the mouth (pTis pN0 M0) diagnosed in July 1999 and surgically treated with tumor exeresis, functional bilateral supramilohid lymphadenectomy, reconstruction with microvascular free radial flap and tracheostomy. In the postoperative period a cervical hematoma appeared, which had to be surgically debrided. Furthermore, the patient received internal radiation with brachytherapy (total dose 50 Gy).

In 2000, a second OSCC arising in the soft palate and latero-cervical area (pT1 pN2b M0) was detected and treated by local excision with direct repair and a radical lymphadenectomy. No complications appeared during the postoperative period. A second radiotherapy with external radiation with a total dose in the tumor site of 60 Gy (2 Gy per fraction), 50 Gy (2 Gy per fraction) in the supraclavicular field and 60 Gy in the spinal lymphatic right chains and 50 Gy in the left (2 Gy per fraction) were applied between the years of 2000 and 2001. Mucositis and epithelitis GII grade appeared as toxicity consequences of the radiation.

In 2017, a third OSCC was located on the left buccal mucosa (T2 N0 M0) which was treated with tumor resection and without radiotherapy. The defect was reconstructed with a radial microsurgical graft. A vein thrombosis of the pedicle occurred in the postoperative period, this complication was solved with a new vein anastomosis.

The patient is currently being treated for prostate cancer with external radiotherapy.

In addition, the patient was diagnosed with hypothyroidism caused by the radiation therapy, hypercholesterolemia, hiatal hernia, phlebitis, anxiety disorder, cervical stenosis and angor pectoris. These conditions were controlled with: Simvastatin, Omeprazole, Pentoxifylline, Acetylsalicylic acid and Levothyroxine.

**Figure 1:**
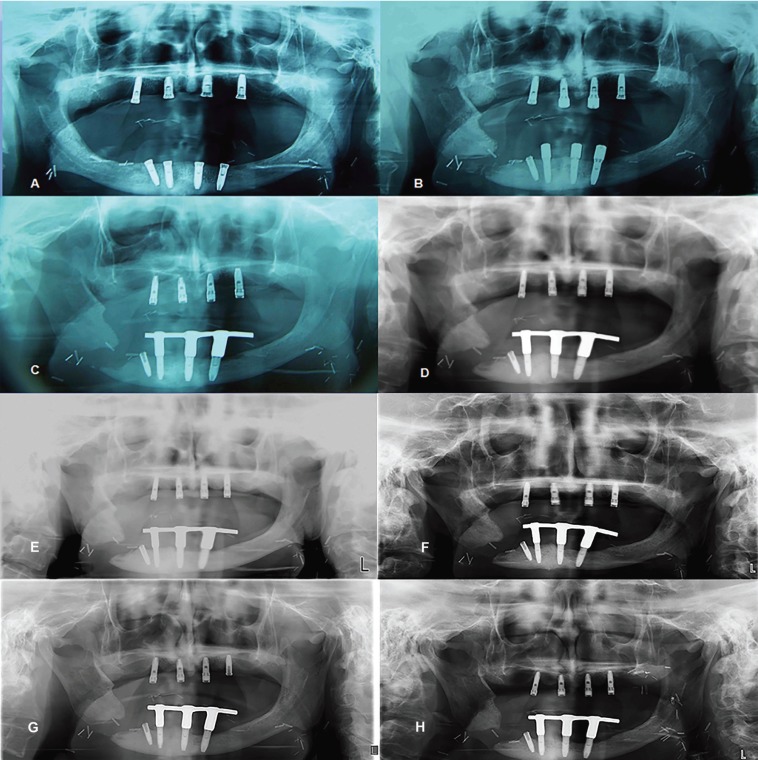
X-ray controls. The mandibular fracture remains separated without an evidence of bone callus formation, over the time. A) December 2008 B) August 2009 C) October 2010 D) October 2011 E) May 2012 F) March 2013 G) December 2015 H) August 2018.

On the first visit consultation in our Department, a panoramic radiography and a computed tomography were taken for treatment planning due to the impossibility of wearing a full prosthesis. Residual alveolar ridge was classified as class III according to the Cadwood and Howell classification. In accordance with the patient, it was decided to insert 4 dental implants in each jaw to support two overdentures. In July 2008, the implant surgery was done (Nobel Speedy groovy® 4.0 mm × 13.0 mm) under local anesthesia with Articaine 4%, 1:100.000 (Ultracain; Normon, Madrid, Spain) and antibiotic prophylaxis (Amoxicillin [GlaxoSmithKline, Madrid, Spain] 2 grams/ 1 hour before) (Figure 1-A). One of the lower dental implants was placed with angulation in order to avoid the emergence of the mental nerve and improve the distance between implants. One week after the surgery, a wound dehiscence with bone exposure occurred on the right side of the mandible. This complication was treated with amoxicillin/clavulanate 875/125 mg [Augmentine 875/125 mg; GlaxoSmithKline, Madrid, Spain] every 8 hours for 10 days, as well as a strict oral hygiene program with 0.12% chlorhexidine digluconate [Clorhexidina Lacer; Lacer, Barcelona, Spain] every 12 hours for 15 days. Two months later an osteoradionecrosis was diagnosed because no total healing was obtained, a surgery with local anesthetic to curette the area was performed but after all the wound kept failing to heal normally.

In March 2009, another curettage with primary closure and a bone biopsy were done under local anesthesia and intravenous sedation. The histopathological study revealed marrow fibrosis without confirmation of bone remodeling or evidence of malignancy. From then on, the wound started to heal normally in the post-operatory period.

**Figure 2:**
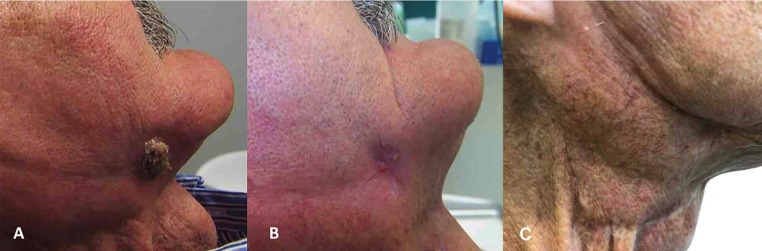
Extraoral fistula: A) August 2009 B) October 2011 C) August 2018.

**Figure 3:**
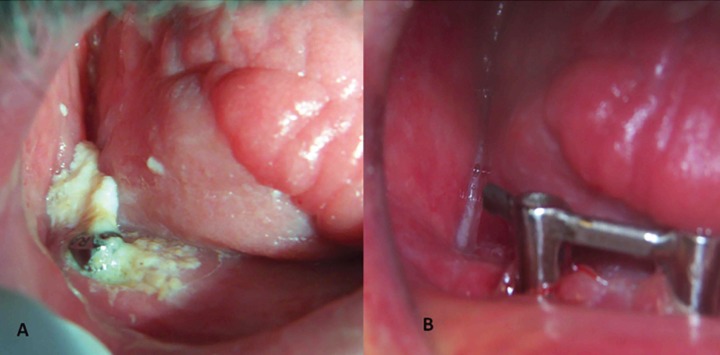
Intraoral wound: A) August 2009 B) Intraoral wound healed October 2011.

In August 2009, the patient was hospitalized due to severe pain and mobility on the right side of the mandible without history of trauma. The clinical and radiological examination revealed a cutaneous fistula in the lower jaw and a mandibular fracture on the right side with 12 mm of distance between bone fragments (Figs. 1B, 2A, 3A). The fistula was debrided twice to remove the purulent content and a soft diet was prescribed along with amoxicillin/clavulanate 875/125 mg (Augmentine ® 875/125 mg; GlaxoSmithKline, Madrid, Spain) every 8 hours for 15 days. The sensibility of the lips and chin were lost as a result of the fracture.

Although several options were suggested in order to repair the fracture (pseudoarthrosis), the patient decided not to be treated. Even though an antibiotic therapy was used immediately after the diagnosis of the fracture, it has not been required since.

A second-stage implant surgery was made after 6 months post-mandibular fracture with Laser Er,Cr:YSGG 1.5 W, 30 pps (Waterlase MD®;Biolase Technology, California, EE.UU.) without complications. Although the implant near to the line of fracture was osseointegrated, it was decided not to use it and an inferior bar supported by the remaining implants and Locator abutments (Ancladen SL, Barcelona, Spain) on the superior ones were used for retaining two overdentures (Fig. 1C). Up to 2017, during more than 8 years of follow-up, the prothesis were functionally useful without significant problems, but since the patient received the radial microsurgical graft his mouth-opening was very limited, and that prevented wearing the prosthesis. Although after 1 year of follow-up, the dentals implants in the upper jaw presented periimplantitis-disease while lower implants exhibited mucositis. Intraoral tissues and fistula on the fracture area were entirely healed at that time period (Figs. 2B, 2C, 3B).

The patient was included in a maintenance program to be controlled radiographically and clinically every 6 months for periodontal maintenance treatment to manage the peri-implant disease but currently did not follow a good fulfillment (Fig. 1D,E,F,G,H).

 Table 1 shows the timeline which summarizes the diagnostics and treatments carried out in our patient.

**Table 1:**
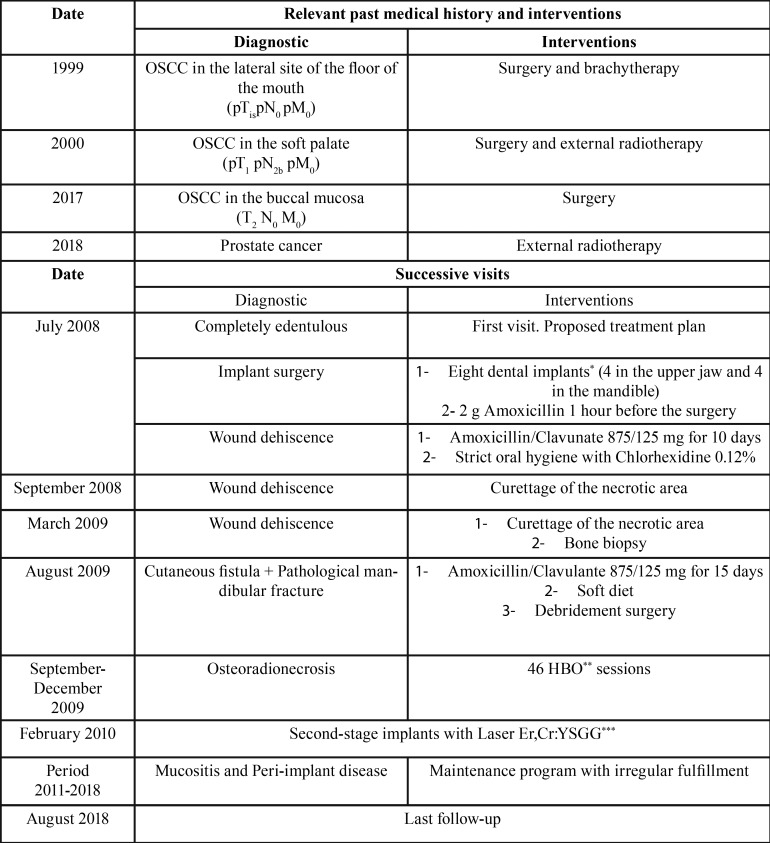
CARE timeline table. OSCC= Oral Squamous Cell Carcinoma. *Nobel Speedy Groovy® (Nobel Biocare, Zürich-Flughafen, Switzerland). **HBO= Hyperbaric oxygen therapy. ***Laser Er,Cr:YSGG (Waterlase MD, Biolase Technology, California, EE.UU).

## Discussion

Radiotherapy is largely used for treatment of head and neck cancer with good results but this treatment has secondary effects which includes orofacial complications, such as changes in mucosa, vascularity, taste, salivary flow, decreased the healing potential, among others (8). These alterations are observed in over 50% of all post-irradiated patients (9).

The alveolar ridge bone may be amongst the most sensitive to systemic alterations in bone metabolism remodeling (10). This is one of the reasons why there has been some controversy on the indication of implant-supported rehabilitations in irradiated patients although several studies have shown that dental implants were not contraindicated in these patients (1,4–6).

The most devastating complication of radiotherapy is ORN, which has been defined as a slow-healing radiation-induced ischemic necrosis of bone with associated soft tissue necrosis of variable extent occurring in the absence of local primary tumour necrosis, recurrence or metastatic disease (8). The diagnosis of this pathology is based on clinical manifestation ranging from occult disease to major bone destruction with soft tissue necrosis or spontaneous complications like pathological fracture (8) as occurred on the patient described which presented a stage three of ORN according to Schwartz and Kagan’s classification (11) but surprisingly away of the area where the dental implants were placed.

Although over the years a variety of treatment modalities have been used in managing mandibular fractures (12), our patient refused to receive any treatment and radiographically the body of the mandible remains completely separated without evident callus formation and without misalignment all over 9 years. This is a quite uncommon event, and we failed to find in the literature any published cases showing similar findings.

Eckert *et al.* (13) reported their experience regarding dental implant placement into irradiated jaws including a case of mandibular fracture as a result of trauma affecting a failed implant site, in which the patient was treated with closed reduction without achieving a bone union, but a fibrous one, probably because ORN is a condition in which cellular activity and collagen formation in bone and periosteum are jeopardized, leading to a hypoxic, hypocellular and hypovascular tissue (8). The mandible seems the most susceptible to this event, due to its vascular pattern, and the buccal surface of the mandible seems to have a worse prognosis compared to similar lesions located in the lingual area (8,14). The mucosa also becomes friable and weak, thus favoring the onset of lesions that fail to heal (9,10) and that is why implant-supported prostheses should be considered instead of tissue-supported ones as there might be less likelihood of soft-tissue trauma. In our patient, this complication was observed in the lingual area of the right side of the mandible body after one week of implant surgery. It had to be noted that during implant placement the incision was done lingualized and failed to close primarily.

Current scientific evidence shows that there are no differences in survival rates when implants are inserted before or after 12 months after radiotherapy (6).

The risk of ORN is chronic and may appear many years after radiotherapy (8). In our patient, the implant surgery was conducted 8 years after radiotherapy under local anesthesia with vasoconstrictor, it has to be admitted that this fact could have been an additional risk for ORN.

There is no consensus treatment for this severe condition. While antibiotics are commonly used for managing ORN, there is not an evidence-based antibiotic therapy that heals ORN (9,14). In our reported case antibiotics, oral antiseptics and debridement surgery were used as a treatment to close the fistula and the intra-oral wound.

Other possibilities focused to achieve the healing of such complication is the use of hyperbaric oxygen therapy (HBO) with the aim to improve the blood supply to the injured tissues but its results remains unclear because sometimes does not offer any appreciable clinical benefits (4,6,14), our patient received 46 HBO sessions in 2009, between September and December .

Pathological fracture of the mandible is the most feared complication in the extremely atrophic mandible, particularly in our case after placement of endosseous dental implants on an irradiated patient. This situation has been barely mentioned in the literature, however it has probably occurred in many unreported cases (15). All published fractures appeared in mandibles with height length of 10 mm or less (Cadwood and Howell Class IV) without osseous pathology. The fracture can occur during implant placement, because of implant failure in atrophic mandibles (3,15) or after surgery as implant placement weakens the already-compromised mandible (3). Furthermore, it seems that the site of an implant yet to be osseointegrated is an area of stress concentration and weakness (10). In our case, neither of these factors have been the cause of the mandibular fracture since the degree of resorption was classified as grade III and no one implant was included in the line of fracture.

Other etiologic factors of fracture reported are: osteoporotic changes (decreased bone mass) that might affect the atrophic mandible, deficient mineralization that might occur after bone graft placement and stress concentration at the implant site during implant placement and the tensile forces that occur within the mandible during normal function (3,12). The risk of fracture in these patients is high because of the loss of proprioceptive sensitivity, which allowed patients to apply excessive occlusal forces, besides the lower jaw is known to be much more vulnerable to tensile forces than to compressive forces (10) . Another important factor is the marginal bone loss around a dental implant, as this also contributes to the fracture (15) as it has been reported in the case of Eckert et al. (13).

Treatment of pathological fractures that are associated with such conditions can be challenging, and it should differ according to etiology.

In conclusion, the treatment and management of oncologic patients is a challenge and special considerations are always required. It is necessary to largely inform the patient about risk factors and complications.

## References

[1] Cuesta-Gil M, Ochandiano-Caicoya S, Riba-Garcia F, Duarte-Ruiz B, Navarro-Cuellar C, Navarro-Vila C (2009). Oral rehabilitation with osseointegrated implants in oncologic patients. J Oral Maxillofac Surg.

[2] Renvert S, Quirynen M (2015). Risk indicators for peri-implantitis. A narrative review. Clin Oral Implants Res.

[3] Chrcanovic BR, Neto-Custódio AL (2009). Mandibular fractures associated with endosteal implants in the atrophic mandible. Oral Maxillofac Surg.

[4] Diz P, Scully C, Sanz M (2013). Dental implants in the medically compromised patient. J Dent.

[5] Vissink A, Spijkervet F, Raghoebar GM (2018). The medically compromised patient: Are dental implants a feasible option?. Oral Dis.

[6] Curi MM, Condezo AFB, Ribeiro KDCB, Cardoso CL (2018). Long-term success of dental implants in patients with head and neck cancer after radiation therapy. Int J Oral Maxillofac Surg.

[7] Gagnier JJ, Kienle G, Altman DG, Moher D, Sox H, Riley D (2014). The CARE guidelines: Consensus-based clinical case reporting guideline development. J Clin Epidemiol.

[8] Chrcanovic BR, Reher P, Sousa AA, Harris M (2010). Osteoradionecrosis of the jaws--a current review--part 1: Physiopathology and risk and predisposing factors. Oral Maxillofac Surg.

[9] Escoda-Francoli J, Rodriguez-Rodriguez A, Perez-Garcia S, Gargallo-Albiol J, Gay-Escoda C (2011). Dental implications in oral cancer patients. Med Oral Patol Oral Cir Bucal.

[10] Mason ME, Triplett RG, Van Sickels JE, Parel SM (1990). Mandibular fractures through endosseous cylinder implants: Report of cases and review. J Oral Maxillofac Surg.

[11] Schwartz HC, Kagan AR (2002). Osteoradionecrosis of the mandible: Scientific basis for clinical staging. Am J Clin Oncol.

[12] Nasser M, Pandis N, Fleming PS, Fedorowicz Z, Ellis E, Ali K (2013). Interventions for management of mandibular fractures. Cochrane Database Syst Rev.

[13] Eckert SE, Desjardins RP, Keller EE, Tolman DE (1996). Endosseous implants in an irradiated tissue bed. J Prosthet Dent.

[14] Chrcanovic BR, Reher P, Sousa AA, Harris M (2010). Osteoradionecrosis of the jaws--a current review--part 2: Dental management and therapeutic options for treatment. Oral Maxillofac Surg.

[15] Boffano P, Roccia F, Gallesio C, Berrone S (2013). Pathological mandibular fractures: A review of the literature of the last two decades. Dental Traumatology.

